# Supplementary material for: The causal relationship between the human gut microbiota and pyogenic arthritis: a Mendelian randomization study
Source: Front Cell Infect Microbiol. 2024 Nov 26;14:1452480. doi: 10.3389/fcimb.2024.1452480 (PMC11629706; doi:10.3389/fcimb.2024.1452480)
Supplement: Supplementary file 1 [file DataSheet1.zip › mendelian test/ebi-a-GCST90027537.csv_leave_one_out.pdf]

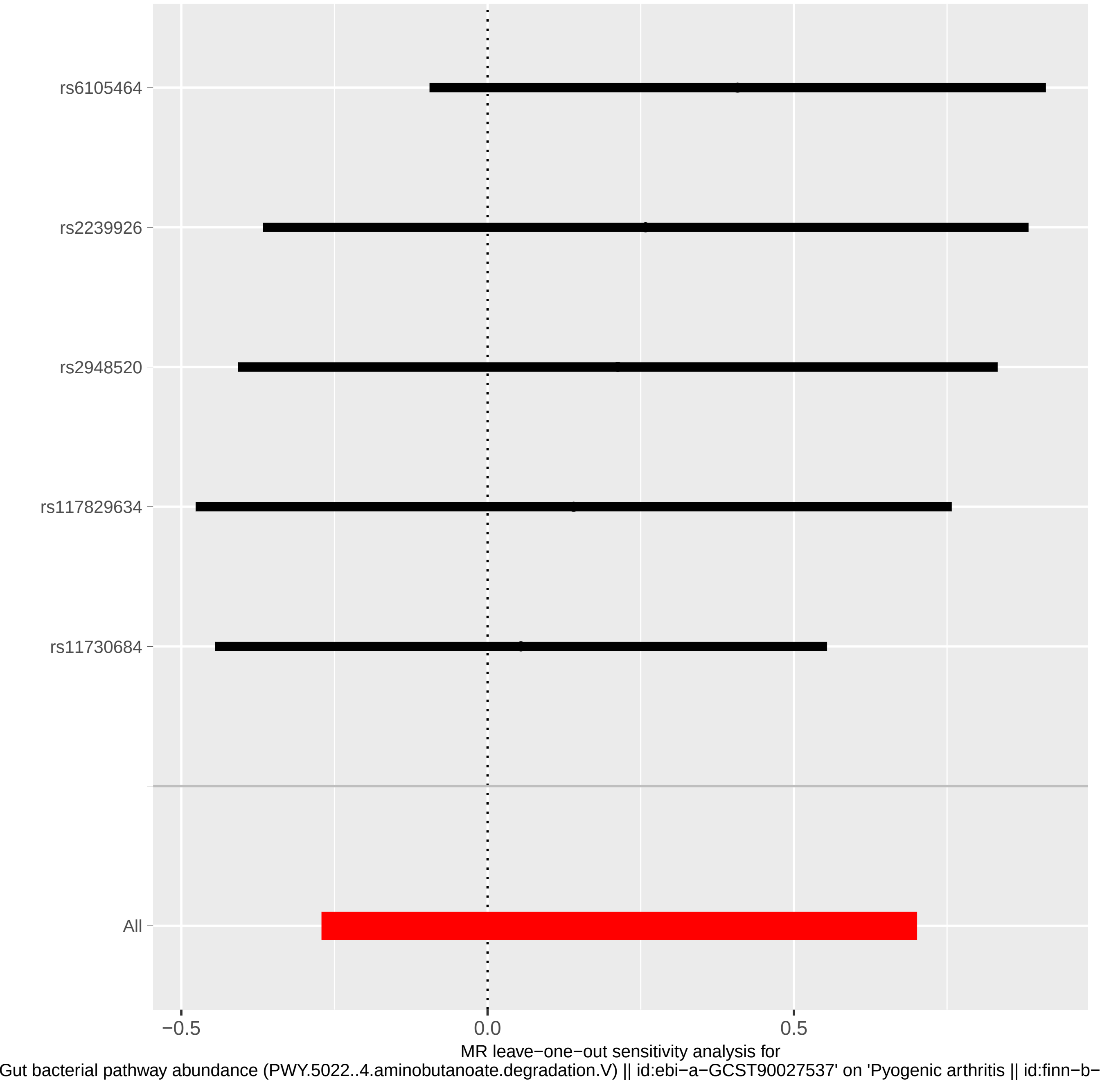

rs6105464
rs2239926
rs2948520
rs117829634
rs11730684
All
−0.5
0.0
0.5
MR leave−one−out sensitivity analysis for
Gut bacterial pathway abundance (PWY.5022..4.aminobutanoate.degradation.V) || id:ebi−a−GCST90027537' on 'Pyogenic arthritis || id:finn−b−
